# Palmar pseudoaneurysm following carpal tunnel release

**DOI:** 10.1080/23320885.2025.2468438

**Published:** 2025-03-04

**Authors:** Sydney Bormann, Tiffany Bender, Benjamin Kulesa, Jason Fowler

**Affiliations:** a University of Kansas School of Medicine, Sioux Falls, SD, USA; b University of South Dakota Sanford School of Medicine, Sioux Falls, SD, USA; c Avera Medical Group Plastic and Reconstructive Surgery, Sioux Falls, SD, USA

**Keywords:** Pseudoaneurysm, carpal tunnel

## Abstract

Carpal tunnel release surgery is a common and relatively safe surgical procedure; however, rare complications such as pseudoaneurysms can occur. Treatment of palmar pseudoaneurysms typically includes endovascular embolization or open ligation. We present a case of open surgical excision and repair of a pseudoaneurysm of the superficial palmar arch following carpal tunnel release surgery. This case study demonstrates how common surgical procedures can yield serious complications, thus highlighting the importance of thorough evaluations in pre-and post-operative care.

## Introduction

Carpal tunnel syndrome is a very common peripheral neuropathy and a frequently encountered disorder of the hand [1,2]. Carpal tunnel release is the standard procedure performed to alleviate compression on the median nerve by increasing the volume of the carpal tunnel through division of the transverse carpal ligament [3]. Complications, although rare, can include intraoperative technical errors, postoperative infection or pain, and persistent or recurrent symptoms, all of which have a relatively low incidence of occurrence [4]. The most severe complication of carpal tunnel release includes injury to a nerve or artery. Pseudoaneurysm of the superficial palmar arch is a rare neurovascular complication, with iatrogenic damage to the structure occurring in 0.1% of carpal tunnel release operations [1]. The majority of these injuries develop due to traumatic injury to the hand or ligation of an artery during surgery and usually only manifest after the surgical site is closed and the tourniquet is removed [1,5,6]. A pseudoaneurysm or false aneurysm arises when the vessel wall is disrupted and the surrounding tissues contain a resulting hematoma [7]. Diagnosis is made based on high clinical suspicion after evaluation, especially in the presence of a pulsatile mass within the palm. The treatment of these lesions is a matter of debate due to the variation of each patient’s case, but commonly chosen solutions range from minimally invasive endovascular embolization to more invasive open ligations, which involve complex microvascular surgery, open thrombectomy, and/or transversal arteriotomies [6,7]. This case study demonstrates how even common surgical procedures can yield serious complications, thus highlighting the importance of thorough evaluation in pre-and post-operative care [8].

## Case report

An 88-year-old male with a past medical history significant for pulmonary embolism, essential hypertension, and long-term anticoagulant use presented with an enlarging left hand mass with distal weakness and paresthesia four months following an open carpal tunnel decompression with tourniquet application. Surgery was without complications and anticoagulation was resumed after surgery. Three days after surgery, the patient was seen by his primary care physician for increased edema of the left upper extremity. One week after surgery, he was seen in follow-up with orthopedic surgery, where ecchymosis and edema were noted. Three weeks following surgery, he was seen by primary care for worsening discomfort and edema. Over the following two months, he continued to experience left hand pain and increased numbness in his third and fourth digits. On examination, he had tenderness to palpation and increased swelling of the hand and wrist. A hematoma was suspected and an MRI was ordered.

**Figure 1.**
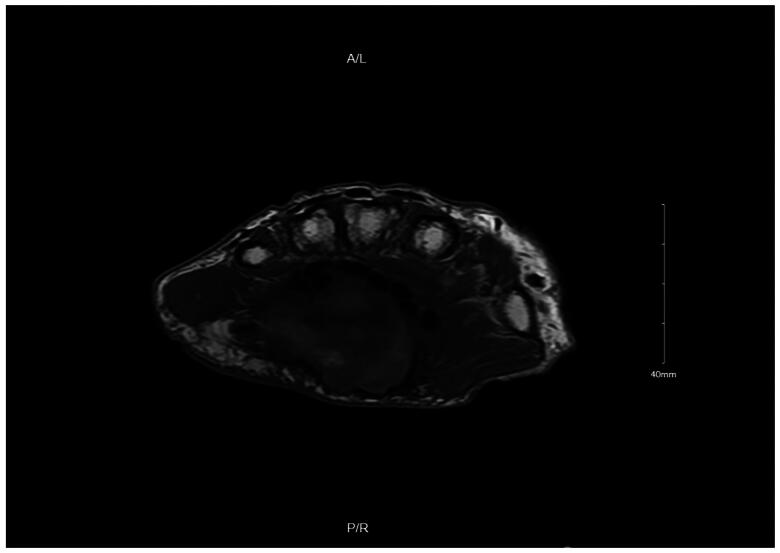
Coronal MRI of the left hand.

**Figure 2.**
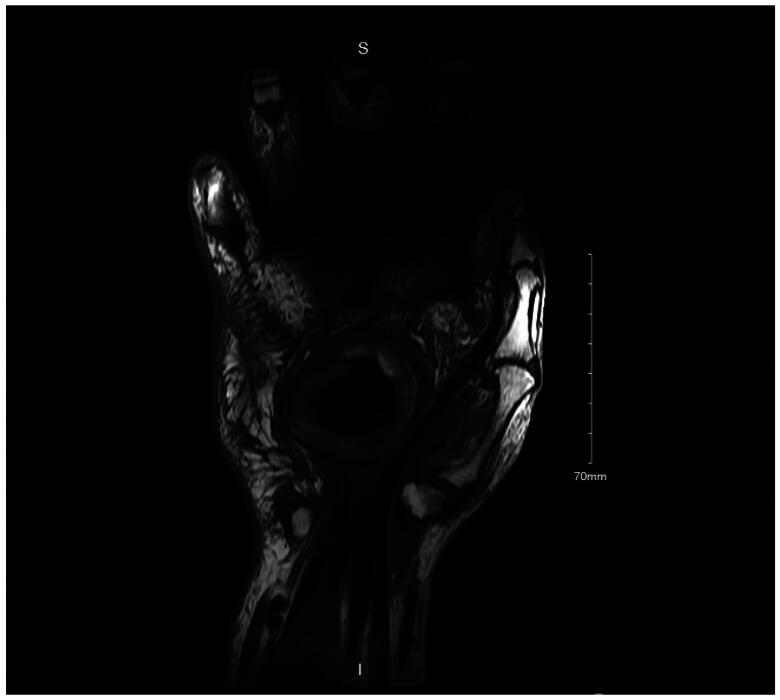
Axial MRI of the left hand.

An MRI (Figures 1 and 2) was obtained 14 weeks following surgery and revealed a complex well-circumscribed mass within the palmar side of the left wrist at the level of the flexor retinaculum superficial to the flexor tendons. The mass measured 4.7 × 2.9 cm transversely and 4.3 cm craniocaudally. The mass contained a mixture of high and low signal intensity material on T1 and T2 weighted images. On T1 and T2 weighted images, an irregularly shaped central area of markedly low signal was present, and a fluid-fluid level appeared to be present. A hematoma was suspected; however, it was not felt to be enhancing as would be expected from a pseudoaneurysm. T1 precontrast fat-suppressed imaging was not performed for direct comparison.

**Figure 3.**
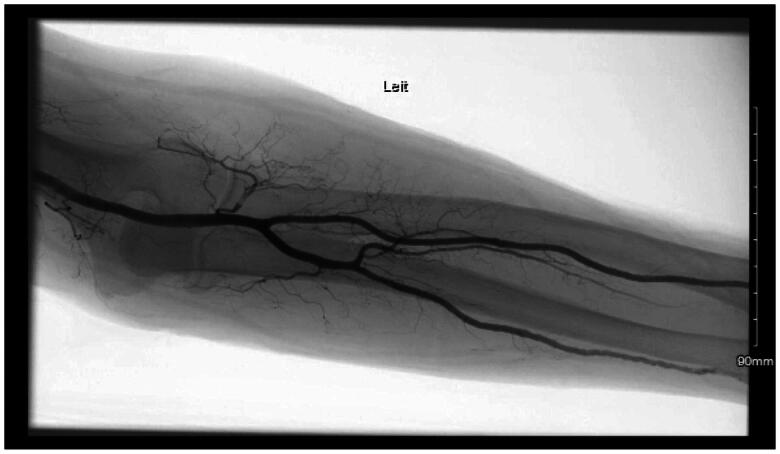
Angiography demonstrating patent arteries.

**Figure 4.**
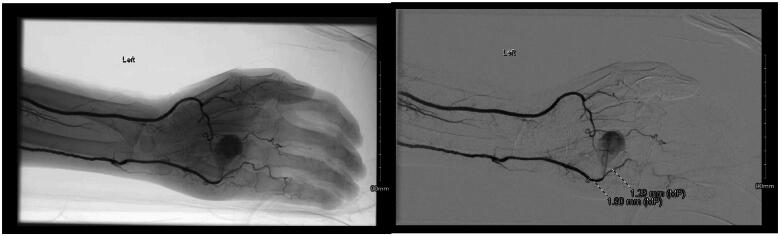
Angiography demonstrating pseudoaneurysm arising from the ulnar artery.

Sixteen weeks after the initial procedure, a CTA (Figures 3 and 4) was performed to rule out a pseudoaneurysm. CTA revealed a 5 cm area of mixed attenuating soft tissue fullness involving the palmar aspect of the left hand at the level of the proximal metacarpals, compatible with blood accumulation. There was no appreciable enhancement. Calcified plaquing of the radial and ulnar arteries and the palmar arterial arch was identified. There was significant suspicion for pseudoaneurysm and additional angiography was recommended.

Completion of left upper extremity angiography was performed. This demonstrated patent brachial, ulnar, radial, and interosseous arteries. A large broad-necked pseudoaneurysm arising from the ulnar artery at the level of the fifth metacarpal body was identified. The ulnar artery more distally supplied the artery to the fifth digit. The radial artery supplied blood flow to the first through fourth digits. Given the broad neck of the pseudoaneurysm, it was not amenable to percutaneous intervention.

The patient was then referred to plastic surgery for further evaluation. He complained of left hand swelling and numbness which had significantly worsened over the last few months. His symptoms were most bothersome in the third and fourth digits and his fifth digit was minimally affected. He was also experiencing left hand weakness, paresthesias, and sensitivity to temperature change.

Physical examination demonstrated a mass in the left central palm with a well-healed palmar scar from open carpal tunnel release. The mass was firm and tense with a strong triphasic Doppler signal and a palpable pulse on the radial aspect of the lesion. Allen’s test was performed using a hand-held Doppler for evaluation of the radial digital artery of the fifth digit. With very minimal occlusion of the ulnar artery, there was complete loss of signal to the fifth digit finger. The digital artery to the fourth digit on the radial aspect remained with Doppler signal on compression of the ulnar artery but was absent with radial artery compression at the wrist. Sensation was significantly diminished to the second, third, and fourth digits, and distal weakness was present. The fingers were well-perfused distally with appropriate capillary refill time.

Given the pseudoaneurysm originated from the ulnar artery and dissected towards the central portion of the palm with a wide base, it was not amenable to percutaneous intervention. Thus, ligation and excision was planned. It was discussed with the patient that reconstruction of portions of the palmar arch may be necessary. Surgery included removal of the aneurysm, isolation, and identification of each of the digital vessels as well as common digital nerves, carpal tunnel release with median nerve neurolysis, and ulnar nerve release.

The patient was taken to the OR and placed in the supine position. Doppler was performed prior to incision. Signals at the digital vessels to the third and fourth digits were not identified. Signal was obtained at the fifth digit radially and the fourth finger ulnarly. The Doppler signal at the aneurysm site was intermittent.

**Figure 5.**
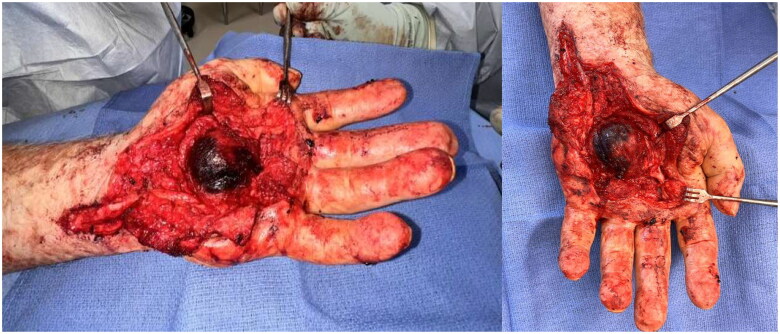
Pseudoaneurysm of the superficial palmar arch.

A Bruner’s incision was made towards the fourth webspace. The incision was opened, and a pseudoaneurysm capsule in the central palm was identified (Figure 5). Meticulous dissection was performed around the volar aspect of the aneurysm. There were many very thin-walled spots with significant clots and dissection was continued both distally and proximally. The aneurysm appeared to enter within Guyon’s canal. The carpal tunnel was released with a z-plasty incision over the wrist crease. The median nerve was isolated and appeared edematous. Neurolysis of the median nerve was performed and the transverse carpal ligament was released. Guyon’s canal was released and the ulnar artery with the corresponding vena comitantes was isolated. A portion of this pseudoaneurysm was reached ulnarly. There was a significant clot distally and the nerves were not easily identified. The Bruner’s incision was therefore extended over the central portion of the proximal third finger and skin flaps were made both radially and ulnarly, identifying the common neurovascular bundles to the second, third, and fourth webspaces. The digital nerves were circumferentially dissected and separated from the wall of the pseudoaneurysm and surrounding clot towards Guyon’s canal. The common digital nerves to the second and third webspace were unable to be identified within the aneurysm distally.

Dissection was continued over the anterior aspect of the aneurysm and the superficial radial contribution to the radial arch was encountered. A clot was visualized within the distal aspect of the arch, but there was no communication proximally, indicating possible traumatic tear during aneurysm expansion. There was no bleeding from the proximal margin and no Doppler signal was identified at the proximal digital vessels. However, the fingers remained perfused distally. Meticulous dissection was then performed and the wall of the pseudoaneurysm was opened. Dissection was performed along the arch distally to the second and third webspace separating the pseudoaneurysm wall from the common digital vessels.

Dissection was performed on the undersurface of the aneurysm, which was densely adhered and interdigitated between the superficial flexor tendons. Flexor tenolysis of the second, third, fourth, and fifth digits was performed, dissecting the aneurysm free from the superficial flexor tendons. Dense adhesions to the thin pseudoaneurysm wall were inadvertently punctured and brisk arterial bleeding was encountered, which could not be easily controlled, requiring tourniquet application. The remainder of the pseudoaneurysm was dissected.

**Figure 6.**
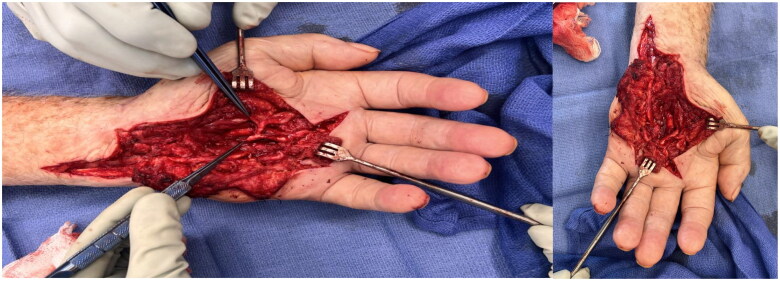
Palm following removal of pseudoaneurysm with identification of an intact branch of the radial artery.

It was unclear where the pseudoaneurysm had finished its dissection. The arch was then divided and transected at the area where there was no clear communication seen between the aneurysm and the inner wall of the vessel. A sidewall was created radially to the takeoff of the common digital vessel to the fourth webspace, which was repaired with multiple small gem clips along the radial border attempting to keep the lumen patent. The clot and the aneurysm were then removed (Figure 6).

**Figure 7.**
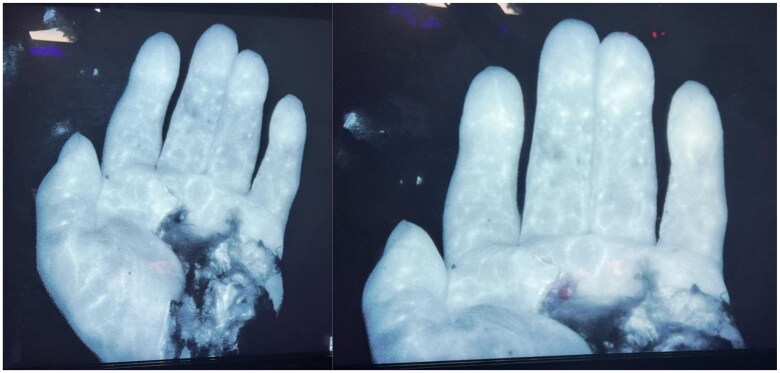
SPY-PHI Imaging demonstrating perfusion to digits.

The tourniquet was then let down and the sidewall repair of the ulnar artery had brisk arterial bleeding. The walls of the pseudoaneurysm around what was the radial extension of the superficial palmar arch were isolated and additional gem clips were placed. The hand was allowed to perfuse for approximately five minutes and indocyanine green was injected. SPY-PHI imaging evaluation was performed and each of the fingers showed signs of perfusion despite the absence of radial flow through the superficial palmar arch. The ulnar vessel could be visualized providing perfusion to the common digital vessel to the fourth webspace, perfusing the fourth and fifth digits (Figure 7).

It is likely the contributions from the palmar arch distally either through the second or third webspace were communicating with those digital vessels providing flow to the second and third finger. This was monitored both by Doppler and by SPY to ensure sustained flow. The ulnar vessel was assessed via Doppler and showed a strong pulse of the common digital vessel. The digital vessels on the radial and ulnar aspect of the second and third digits were detectable only distal to the webspace despite having well perfused fingers.

Adequate hemostasis was performed and the incision was closed. The hand was again evaluated, and the digits were well-perfused with adequate capillary refill and turgor. A volar splint was placed, and the aneurysm was sent for pathology. There were no intraoperative complications. The patient’s hand was examined after emergence from anesthesia and remained well perfused.

The patient returned for follow-up on postoperative day three. He was recovering well with minimal pain. He had persistent numbness in his left first, second, and third digits as well as sensitivity to cold in all digits of his left hand. His pain and discomfort had overall improved since surgery. On examination, range of motion of his second, third, fourth, and fifth digits were limited for flexion, and there was desquamation of the palm centrally.

**Figure 8.**
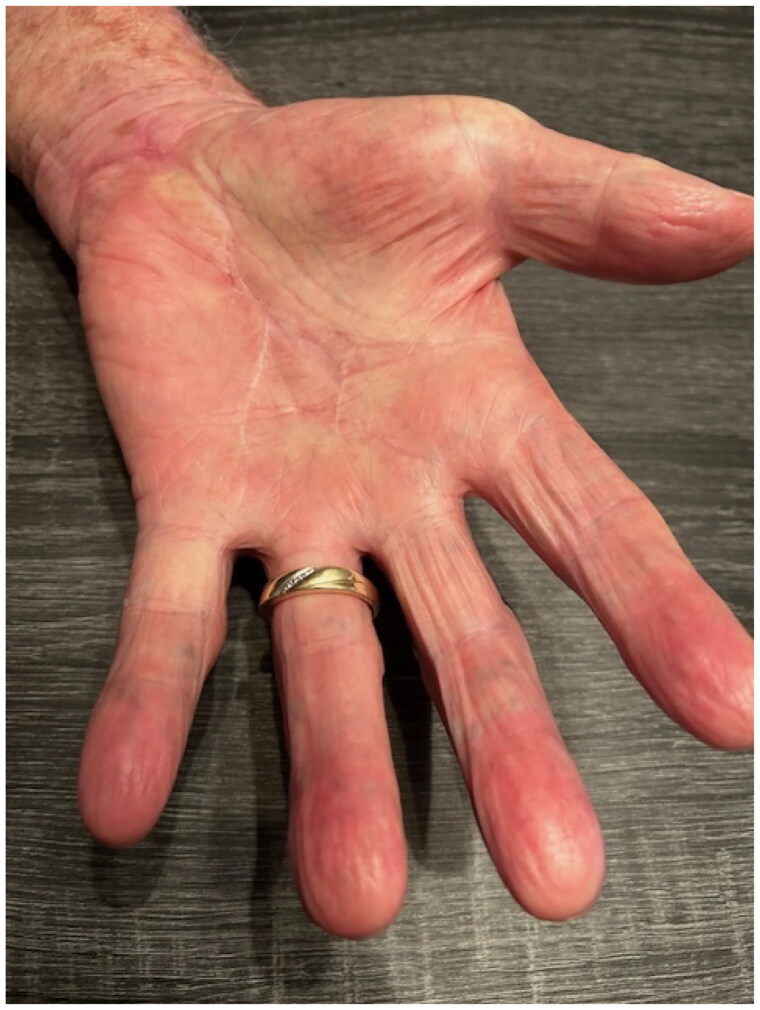
Palm seven months following surgery demonstrating a well healed scar and distal perfusion.

On postoperative day seventeen, the patient returned for follow-up. A 2 × 1.2 cm area of hypertrophic granulation tissue was present on the palm. Excisional debridement of the skin and subcutaneous tissue was performed. The patient has otherwise healed well with improvement of range of motion and resolution of pain (Figure 8). Objective testing, such as grip strength and DASH scoring, was not pursued as the patient resided several hours away in a rural area and self-reported he had returned to his activities of daily living (ADLs), deeming further in-person testing unnecessary.

## Discussion

Surgically induced neuropathic pain is a serious clinical problem, with an estimated 10-50% of all patients experiencing it after common operations [9]. It is very prevalent, yet difficult to resolve due to the marked variability in patient responses to identical surgical procedures as a result of their individual factors such as age, gender, genetics, preexisting pain, and past medical history [8,9]. During carpal tunnel release the median nerve is the center of focus as it has two branches within the vicinity of the carpal tunnel that are at risk of injury during operation.

Although peripheral nerve injuries are the primary complication of carpal tunnel release, arterial injuries are also prevalent and can cause significant harm to the patient if not identified. Pseudoaneurysm of the superficial palmar arch is a rare entity with few cases discussed in the literature, often leading to a delayed or inaccurate diagnosis [5–7]. This is combined with the anatomical intricacy of the structure as it lies just distal to the transverse carpal ligament and is obscured in fat, making it vulnerable to injury [1]. While the patient presented in this case report did not have an anatomic variation, it is important to recognize anatomic variations in the palmar arch exist. The superficial palmar arch can be described as ulnar artery dominant, radial artery dominant, or codominant with equal blood supply from the radial artery and ulnar artery [10]. The superficial palmar arch can also be described as complete or incomplete depending on the presence or absence of blood supply to the thumb [10]. Although carpal tunnel release is generally a low risk operation, care should be taken during surgery to properly visualize all structures and relevant anatomy to prevent potential pseudoaneurysm formation.

The differential diagnosis is complicated by the anatomy and rarity of pseudoaneurysms, as well as diagnosis of exclusion from fibromas, incision cysts, and lipomas [5,6,11]. Secondary complications of a pseudoaneurysm include infection, formation of an abscess, arterial occlusion, and nerve compression [5]. The presence of a pulsatile mass is a primary sign of a false aneurysm, with increasing concern if the mass progressively arises [1]. Such pulsation also differentiates an aneurysm from a hematoma.

Preference of initial imaging is dependent on patient presentation. In the absence of major concerns for ischemia or loss of sensation, ultrasound is a reasonable initial workup. Ultrasound is able to differentiate between a hematoma and an aneurysm by demonstrating blood flow, or lack thereof, using Doppler. X-ray and MRI are often performed to exclude the presence of a foreign body and assist in initial diagnosis. Angiography is often used in confirming the diagnosis and delineating the origin of the pseudoaneurysm as it can specify the exact location of injury, arteries with sufficient and insufficient flow, and the presence of collaterals which can be useful in planning reconstructive surgery [6]. Some reports also suggest the use of ultrasound is equally as effective as angiography; however, MRA is considered superior in diagnosing pseudoaneurysms [5,7,12]. Overall, evaluation should include a detailed history and physical examination in addition to imaging and possibly electrodiagnostic examination. Evaluation should also include nonoperative management including splinting, injections, occupational therapy, and desensitization.

The blood supply to the hand is primarily formed from an anastomosis between the ulnar and radial arteries which coalesce to form the superficial and deep palmar arches [1,3,5]. The superficial palmar arch exhibits high anatomical variation. It is primarily supplied by the ulnar artery and 25.5-37% of the population have no contribution from the radial artery [5,12]. Pseudoaneurysms in the hand are extremely rare in the adult population with more complications seen in men possibly due to increased subcutaneous tissue in the operative field [4]. Generally, pseudoaneurysms occur due to penetrating hand trauma, repeated blunt trauma, connective tissue diseases, infection, congenital defects, and vasculitis [5]. The primary mechanism through which pseudoaneurysms form is disruption of the arterial wall resulting in bleeding and formation of a hematoma which is later surrounded by fibrous tissue, creating a pocket with the artery [5,7]. In this case, the enlarging hematoma applied pressure to surrounding nerves resulting in numbness and tingling, while the loss of blood flow from the ulnar artery to the superficial palmar arch resulted in the patient’s symptoms in the third and fourth digits. The blood supply to the arch from the radial artery sufficiently vascularized the second and third digits, minimizing symptoms.

The treatment of a pseudoaneurysm in the hand varies from patient to patient due to anatomical variance of the vessels, lesion location and severity, and adequacy of blood supply from collateral vessels. Although surgery is generally required, conservative methods such as compression bandages can adequately treat small, uncomplicated, fully thrombosed pseudoaneurysms, but may be dangerous due to the risk of distal embolization with subsequent occlusion of runoff arteries [6]. Another less invasive route is through endovascular coil embolization which involves the use of a flexible wire inserted into the vessel which fills or closes the vessel to prevent bleeding or rupture of the aneurysm [6]. This procedure is generally favored in cases where the aneurysm does not prevent circulation. Ultrasound-guided thrombin injection has also been found to be an effective treatment of pseudoaneurysms that has been shown to be safe and time-saving [5]. Surgery is commonly the treatment of choice for pseudoaneurysms of the superficial palmar arch to restore blood flow to the digits. The excision and vascular reconstruction are often performed to ensure distal perfusion and avoid complications associated with less invasive treatments [5]. The operation generally requires open surgical resection with ligation of the artery and reconstruction with end-to-end anastomosis [6]. Despite the frequency and relative safety of carpal tunnel release surgery, rare complications such as pseudoaneurysms can occur, which cause severe symptoms and prove difficult to diagnose and treat.
